# Progress in Medicine

**Published:** 1897-08-28

**Authors:** 


### Progress in Medicine.

### RENAL MEDICINE.

Vissman[1] holds that the various forms of Bright's disease are, in the long run, all due to lesions of the alimentary tract. Thus in cholera and the summer diarrhœa of children, where the epithelium of the canal and its secretions are disorganised, we often find various grades of kidney disease. In some cases the secretions of bacteria, in others the germs themselves, enter the circulation and affect the kidneys. Many persons, too, have slight digestive troubles which lead to arterial degeneration and sometimes to severe renal lesions. The rapidity of elimination of drugs and poisons taken by the mouth varies with the kind and quantity of food taken. Thus, according to D. Mokriewitsch,[2] it is more rapid with a meat diet than with a vegetable, quicker with a full diet than with a spare one, and very slow during fasting. It will be remembered that most authorities hold, however, that great uncertainty exists as to the true rapidity of elimination, so much so that the salol test for the motor power of the stomach has been largely disused. Bernhardt,[3] too, believes that the poisons of the acute infectious diseases are the usual causes of kidney disease. In children granular degeneration starts with lesions of the glomerali, though the symptoms are the same as in adults.

With reference to granular kidney, the presence of retinitis has been generally and rightly regarded as a most grave symptom, but Hinshelwood[4] points out that when the renal changes are mainly parenchymatous a more favourable view is possible. He recently brought forward a patient who was attacked with nephritis, dropsy, and uræmia towards the end of 1893. In the next year double optic neuritis with partial loss of vision followed, but since then the ophthalmoscopic appearances have gradually diminished and the albumen has vanished from the water. In the discussion which followed his paper another case was described in which the patient recovered after puerperal albuminuria, but with impaired vision. Hæmorrhages sometime occur in the ears and from the nose during the early stages of diseases of the kidney, and are of unfavourable prognosis. They should draw attention to the possibility of such a disease being present. Haug[5] records a case where epistaxis, followed by bleeding into the ears and cardiac irregularity, formed the first symptoms of an outbreak. He thinks these occurrences are often overlooked, and that the bleeding takes place by diapedesis rather than by rupture of the vessels. With regard to treatment of high tension, if this is actually present, Maguire[6] considers that the best results are obtained by giving frequent saline purges, with periodical mercurial ones, the latter being of great value, and by moderating the amount of nitrogenous food. Lastly, from time to time, while he reduces the arterial constriction he gives strophanthus and strychnia to support the heart and prevent degeneration. In the course of his paper he mentions a unique instance of the prolongation of life for more than seven years after albuminuric retinitis had set in. There had been very high tension, but death occurred finally from influenza. The value of milk diet in chronic disease has, he thinks, been much over-estimated. Ferran[7] agrees with him, and would give a vegetable and fish diet, (*a*) when the urine exceeds a certain limit; (*b*) when the urea and phosphates are increased; (*c*) when the albumen is not excessive, nor the granular casts very numerous; (*d*) and when the general condition is satisfactory.

A most important review of the pathology of the so-called Bright's diseases has appeared from the pen of

W. T. Councilman.[8] The diffuse lesions are, he holds, produced by soluble poisons carried to the kidney by the blood, but their classification must depend for the present on the anatomical lesions, because of our scanty knowledge of the bacterial and other poisons concerned. He proposes the following divisions: A. Acute diffuse nephritis: (*a*) Degenerative. This occurs in fevers, anæmia, and from the action of poisons, and presents chiefly changes of the epithelium of non-inflammatory character; (*b*) glomerular, found in diphtheria endocarditis, measles, and without other disease; (*c*) hæmorrhagic; (*d*) interstitial non-suppurative, also found in diphtheria, scarlatina, and typhoid. B. Subacute glomerular nephritis. C. Chronic: (*a*) Glomerular; (*b*) arteriosclerotic; (*c*) interstitial. D. Senile nephritis from disease of the vessels, with failure of regeneration. E. Amyloid nephritis. In accordance with this grouping he describes the results found in fifty-two postmortems. The bacteria were usually part of a general septicæmia, the pneumococcus being the most frequent, and the streptococcus next. The pathology of each type of disease is described with great fulness.

The production of uræmia and its causes are the subject of investigations by C. A. Herter,[9] by which it is shown that uræmia may be produced without any increase in the urea contained in the blood. The blood, however, is always more toxic, as shown by injecting the serum obtained from uræmic cases into animals, and in some instances there is an increase of the urea. Still, the latter is not necessary for the production of uræmia, as less than the normal amount may be found. Its accumulation in the blood may, however, produce diarrhœa and vomiting. Fatal effects are only produced by doses of ten or fifteen times the amount usually excreted daily. The toxic agents in uræmic blood are in many instances lessened or destroyed by heating; beyond this we know nothing of their composition, but they are neither extractives of the blood, nor of the urine, nor are they potassium salts nor ammonium carbonate. In persistently febrile uræmia there is some infection present. Thus we get three types of the disease—(*a*) pure toxic uræmia, which is rare; (*b*) that from retention of urinary constituents; and (*c*) uræmia from combined bacterial and autogenous toxins, which is the most common. The affection is not a pathological entity, but arises from various poisons which damage the kidneys.

In the discussion which followed the reading of Herter's paper, Draper and Jacobi emphasised the danger of so-called uræmic conditions arising in infectious diseases and from intestinal absorption. The existence of intestinal putrefaction is often estimated by the amount of indican in the urine; but Coffey[10] finds that the amount of indican is no safe measure of the organic sulphates which arise from the putrefaction of proteids. These form one-tenth of the sulphates in normal urine, but are greatly increased when much putrefaction has gone on. He would estimate them by Salkowski's method instead of trusting to the indican only.

Potain[11] has reported a case, unique in his experience, which he considers to be one of acute miliary tuberculosis, chiefly affecting the kidney. The patient had suffered from a salpingitis, probably tuberculous, and the urine was now that of acute nephritis, blood-stained, and containing both albumen and casts, but there was no œdema. He found slight dulness and altered respiratory murmur in the lungs, but there was no cystitis or pyelo-nephritis to indicate an ascending lesion of the kidneys, and these organs appeared normal on palpation. Epithelial nephritis occurs but rarely in tuberculous affections; still, an instance has been noted by Pousson where the primary lesion of miliary tuberculosis was in the kidneys, the other organs remaining healthy until a late period. Such cases must be distinguished from the common, slowly progressive, more or less unilateral tuberculous disease of the kidneys. Another little-understood form of renal disease is polycystic degeneration. Höhne[12] made the diagnosis of this affection from certain rosette-like bodies found in the fluid obtained by puncture. These have been previously described by Beckman and others, but have been overlooked by many writers on the subject, though of some importance as a means of distinguishing this obscure disease. They are only contained in the brown as distinct from the blue and yellowish cysts, but their appearance is characteristic. Under a power magnifying about 300 times, they appear to be of a size varying from a lentil to that of a sixpence. They are marked by concentric rings and a radiating striation.

[1] Med. Record, Jan. 9. [2] Med. Record, March 20. [3] Med. Week, March 5. [4] Glasgow Med. Journ., April. [5] B. Med. Jour., Feb. 6. [6] Clinic. Jour., Mar. 31. [7] Amer. M. S. Bulletin, Mar. 10. [8] Amer. Jour. Med Sc., Jan. [9] Med. Rec., Mar. 21. [10] B. Med. Jour., March 13. [11] Med. Week, Jan. 29. [12] Med. Chron., Jan.

